# Relationship between job involvement, perceived organizational support, and organizational commitment with job insecurity: A systematic literature review

**DOI:** 10.3389/fpsyg.2022.1066734

**Published:** 2023-01-11

**Authors:** Chin Ling Hngoi, Nurul-Azza Abdullah, Wan Shahrazad Wan Sulaiman, Norshaffika Izzaty Zaiedy Nor

**Affiliations:** Faculty of Social Science and Humanities, Universiti Kebangsaan Malaysia, Bangi, Malaysia

**Keywords:** job involvement, perceived organizational support, employment flexibility, job insecurity, organizational commitment

## Abstract

This manuscript aims to review the literature on the relationship between job insecurity and job involvement, perceived organizational support, organizational commitment, and positional characteristics. The definition and conceptualization of the variables are discussed for clarity. This systematic review used the Preferred Reporting Items for Systematic Reviews and Meta-Analyses (PRISMA) guidelines to summarize and review 19 articles on job insecurity. The result shows gaps in the relationship between job involvement and organizational commitment, yielding no result from the search. This review identified implications and areas for future research on the topic. It also found evidence supporting the need to further investigate the antecedents and consequences of job insecurity in order to improve productivity and reduce attrition.

## 1. Introduction

In line with the Industrial Revolution 4.0, which is characterized by digitalization, the availability of technology has transformed the way in which businesses and individuals work. In addition, the changes in working styles increased competitiveness, and the outbreak of the coronavirus disease (COVID-19) pandemic has presented many formidable challenges for both organizations and employees. Therefore, organizations are turning to creative strategies such as downsizing, restructuring, and flexible employment to ensure sustainability in the face of volatile global economic situations.

In the past, relocation or cross-cultural management was rare due to the immobility of the workforce. However, with the availability of low-cost airlines within the region, such as Air Asia and Scoots, workforce mobility and cross-country management are becoming more common, particularly in developing countries such as Southeast Asia. For example, Singapore and Hong Kong are becoming common headquarters for corporations to manage their business locally in the region. Hence, organizations are changing their workforce strategies to increase local presence and productivity.

All these changes affected the content and structure of work, either directly or indirectly. The pressure employees face at work is increasing, resulting in uncertainty and job insecurity among employees. The said changes affected graduates; research shows that graduated unemployment increased due to increased demand in the skill sets and according to market niche (Omar et al., 2012; Sarfraz et al., 2018). Apart from the aforementioned macroenvironmental factors, organizational changes also contribute to feelings of insecurity among employees (Shoss, 2017). The level of job involvement and the perceived organizational support become predictors of job insecurity among employees. Job insecurity would negatively affect employees’ commitment and decrease productivity (De Cuyper and De Witte, 2005; Kraimer et al., 2005; Chen et al., 2007; Green and Leeves, 2013; Wilczyńska et al., 2015; Shoss, 2017).

Organizations are interested in exploring the factors that influence their success and how they impact workforce dynamics and productivity, aiming to increase profitability and remain competitive in the industry. Hence, researchers sought to study variables that influence the organization. The relationship between an organization and an employee affects job performance, productivity, and relationships among colleagues, and it also significantly impacts society (Mowday et al., 1982). Industrial and organizational researchers have identified that work attitude affects productivity and employee commitment (Meyer and Herscovitch, 2001; Meyer et al., 2004; Mugizi et al., 2015).

In previous research, job or employee satisfaction was the center of focus (Jones et al., 1998; Hirschfeld, 2000). However, the focus has gradually shifted to studying other attitudinal concepts such as job involvement, perceived organizational support, and organizational commitment. Academics began studying the “organization” concept as early as the 19th century and theorized several factors influencing employee commitment (Mowday et al., 1982). Studies have shown that no single variable is solely responsible for influencing productivity within an organization. Instead, a combination of various factors and attributes contributes to the overall performance and output of the organization (Judge et al., 2017; Shoss, 2017; Salessi and Omar, 2019).

An extensive literature review of over 70 studies about workplace attitudes, including job involvement, perceived organizational support, and organizational commitment, emphasizing the importance of antecedents and predictors that may influence organizational behavior, has been conducted (Rhoades and Eisenberger, 2002). According to the authors, the interaction between employees and other elements such as leadership, interpersonal relationships, company culture, and the dynamic of the relationship could play a role in shaping organizational behavior.

The concept of job insecurity can be traced back to the early 1990s, and its impact on employees has been studied (Roskies and Louis-Guerin, 1990; Manski and Straub, 2000). Job insecurity is identified as one of the most critical aspects of work quality. In Malaysia, the Industrial Relations Act of 1967 (2012) and the Employment Act of 1955 (2017) provide legal protection for workers from being unfairly terminated or forced to leave their organization. These acts safeguard employees’ interests and oversee organizations’ termination procedures to ensure that employees’ job security is maintained. Employees could file a report at either the Labor Department or Industrial Relations Department should they feel they are being mistreated or unfairly dismissed.

Job insecurity presents an invisible problem for organizations. Employees must juggle day-to-day work while battling the uncertainties of job retention, which is psychologically taxing and also affects organizational outcomes. Employees expend finite resources to complete their work tasks and address any potential threats to their job security. The increase in potential workload is a source of anxiety for employees, which reduces job satisfaction and acts as a distraction that negatively impacts individual performance and organizational productivity (Greenhalgh and Rosenblatt, 1984; Jones et al., 1998).

Organizational commitment is considered one of the most significant work attitudes in the study of organizational and industrial psychology (Allen, 2001; Llobet and Fito, 2013; Wan Sulaiman et al., 2013; Gvpn et al., 2018; Ibrahim et al., 2018). Extensive research in the past four decades indicates that organizational commitment has a significant relationship with various organizational outcomes and employee productivity (Porter et al., 1974; Meyer and Allen, 1997; Chen et al., 2007). Researchers argued that employee commitment is not only influenced by interaction experiences but also affected by the perceptions of organizational support, job and organization stability, and security of employment within the organization (Chen et al., 2007; Shoss, 2017; Salessi and Omar, 2019). Research has shown that apart from the interaction experiences, there were positive relationships and influences between organizational commitment and emotional intelligence (Chow and Abdullah, 2020).

Therefore, organizations must investigate the elements of job insecurity and how perceived organizational support influences it, along with the impact on employee commitment. In addition, it is vital to determine the negative impact of these changes on individual employees and to study management changes and coping strategies for employees to have the ability to cope with potential stress and job burnout due to changes within the organization. These negative impacts have decreased the productivity of the employee and the organization. Apart from work attitudes, demographic variables such as age, gender, educational background, and financial stability play a role in employee social changes within the organization and in daily life.

## 2. Job insecurity

Job insecurity has been conceptualized and defined in several ways. Bordia et al. (2004) view it as a function of objective circumstances, such as contract work that carries a specified term of service. Greenhalgh and Rosenblatt (1984) view job insecurity as a perceptual phenomenon that varies in intensity, even when employees are confronted with job threats. Employment is an important material source of financial stability and personal satisfaction (Vujičić et al., 2015).

Research has shown that job insecurity harms employees’ wellbeing due to the threat of unemployment (De Witte, 1999). Apart from that, De Witte (1999) also concluded that job insecurity contributes to decreases in health and wellbeing. As the employees cannot focus on maintaining their wellbeing, their energy is instead focused on worrying about their job security. In addition to the definition of job insecurity offered by De Witte (1999), other definitions have been proposed by other authors and researchers.

Job insecurity has been presented in two forms: (a) qualitative—worries about losing the job and jeopardizing working conditions, taking a salary cut, or losing an opportunity for career development; and (b) quantitative—referring to the loss of an important job characteristic (Hellgren et al., 1999; Hellgren and Sverke, 2003). Although different authors define job insecurity in different ways, the general understanding is that job insecurity is an individual’s subjective perception.

Job insecurity presents an indirect problem for organizations. It is an internal perception of employees that becomes related to organizational outcomes as employees go about their work while dealing with uncertainties about job retention. Employees do so by consuming resources to both complete their tasks and respond to their perceived job threats. This additional burden of the job causes anxiety for employees, which decreases job satisfaction, and ultimately presents a distraction that may negatively affect performance and organizational productivity (Mowday et al., 1979; Greenhalgh and Rosenblatt, 1984; Sverke et al., 2002).

## 3. Job involvement

The job involvement construct was introduced in 1960. Since then, hundreds of empirical studies have been conducted to identify the characteristics of diverse work settings as a key to enhancing organizational outcomes (Singh and Gupta, 2014; Mendoza, 2019; Salessi and Omar, 2019). However, the literature on job involvement and how it relates to other work attitudes remains unclear (Salessi and Omar, 2019).

For this research, we identified four conceptualizations relating to job involvement in past research (Salessi and Omar, 2019):

(a) the most important job in one’s life;
(b) the employee’s work participation;
(c) the importance of job performance with the subject’s self-concept and self-esteem (Jans, 1982);
(d) the psychological identification of the cognitive state where the work is being done (Kanungo, 1982; Singh and Gupta, 2014).

Four of them share the denominator of the relevance of the work. The concept of psychological identification is adopted in this research.

The term “job insecurity” has faced criticism and skepticism due to its ambiguous nature and the many different meanings that have been attributed to it. Some authors, such as Kanungo (1982) and Blau and Meyer (1987), have argued that considering involvement as participatory work is redundant in the context of work autonomy and participatory leadership. Similarly, seeing it as a focus on work will inevitably overlap with the work-centered structure. Therefore, since 1980, these ideas have not emerged from valid concepts and have been relegated to specific literature (Brown, 1996).

Kanungo (1982) introduces the constructive construct of job involvement to tackle the confusion surrounding job involvement. He concluded that job involvement represented a cultural belief about the job itself and was a descriptive belief based on the ability of a particular job (the current job) to meet the essential needs of an individual.

Although the concept of job involvement has been widely discussed in the literature, there have been relatively few studies that have examined the relationship between job involvement and job insecurity (Shoss, 2017). Most of the current and existing literature focuses on the relationship between job involvement and other variables, such as organizational commitment and job satisfaction, or as mediating or moderating factors (Clay-Warner et al., 2005; Singh and Gupta, 2014; Mendoza, 2019; Salessi and Omar, 2019). Therefore, this paper conducted a systematic literature review to examine the impact of job involvement as an antecedent of job insecurity.

## 4. Perceived organizational support

Perceived organizational support is conceptualized as an organization’s commitment toward its employees (Eisenberger et al., 1986, 2002; Lynch et al., 1999). It refers to employees’ perceptions of support from the organization that the employee received to assist them in completing their day-to-day tasks as well as taking care of employees’ wellbeing and the extent to which the organization values their contributions. Researchers believe that employees develop a commitment to meet the needs of approval, respect, and affiliation and evaluate the benefits of increased workload (Eisenberger et al., 1986; Lynch et al., 1999; Rhoades and Eisenberger, 2002).

Perceived organizational support increases employees’ obligation to help the organization achieve its goals, their emotional commitment to the organization, and the expectation that their performance will be rewarded. Employees develop this belief based on their personal evaluation of the organization’s commitment to the tangible and non-tangible benefits offered to them. Perceived organizational support can lead to positive behavioral outcomes, including improved performance within and outside of one’s role, as well as decreased withdrawal behaviors such as absenteeism and resignation.

Bohle et al. (2018) concluded that perceived organizational support negatively correlates with job insecurity. Employees generally gauge their own value and wellbeing based on the recognition and support they receive from the organization. If they feel that the organization they are working for is not investing in them, it can lead to feelings of job insecurity that do not align with their expectations, particularly during times of downsizing or restructuring (Shoss, 2017; Bohle et al., 2018). Hence, if the perceived organizational support does not align with employees’ expectations, the feeling of betrayal causes the fear of losing the job and is followed by a decrease in organizational commitment (Fried et al., 2003; Bohle et al., 2018). As such, organizations should consider addressing gaps, if any, to increase perceived organizational support and productivity.

Although there was relatively little research on perceived organizational support until the mid-1990s, research on this topic has flourished in recent years. For example, Rhoades and Eisenberger (2002) conducted a meta-analysis of over 70 studies published before 1999. The latest meta-analysis by Kurtessis et al. (2017) showed that 743 studies were conducted on perceived organizational support. In addition, Kurtessis et al. (2017) demonstrated a clear and consistent relationship between perceived organizational support and its predicted causes and consequences.

## 5. Organizational commitment

Organizational commitment has been conceptualized alongside its measurement scales and theoretical basis for over 50 years (Llobet and Fito, 2013). At times, organizational commitment is termed “employee commitment.” Researchers have shown interest in organizational commitment as it is a crucial behavior outcome that contributes to organizational success (Porter et al., 1974; Meyer et al., 1993; Meyer and Allen, 1997; Chen et al., 2007). People develop feelings of attachment toward the organization over time, which is reflected in their commitment to the job.

In 1984, Allen and Meyer undertook an extensive study and empirical evaluation of the impact of organizational commitment on organizational growth (Allen and Meyer, 1990, 1996; Singh and Gupta, 2014). Allen and Meyer (1990) proposed effective, continuous, and normative commitment as the three main components of organizational commitment. Similarly, they believe that commitment is the key that holds individuals to an organization, and the employee is unlikely to look for other opportunities. They stressed that the three components of organizational commitment should be treated as one and not disparately. Affective attachment reflects affective commitment, which refers to an employee’s desire to be part of the team. Normative attachment or commitment refers to the employee’s obligation to be part of the team. Lastly, continuance attachment reflects dedication and refers to employees’ need to be part of the team.

Affective commitment refers to an individual’s emotional and psychological attachment to their organization. Employees’ feelings of competence in the role, along with the organization’s values, culture, and beliefs that align with their personal beliefs, will influence their decision to remain in the organization. Meyer and Allen (1997) claimed that affective commitment is the most beneficial commitment among the three components linked to organizational outcomes: affective, normative, and continuance commitments.

Continuance commitment refers to one’s understanding of the monetary compensation received by remaining in the organization. Certain rewards and benefits are associated with the tenure of a service, such as long service awards, accumulated sick leave, and retirement funds. Besides the monetary rewards, there are non-tangible benefits of being in the organization, like the opportunity for promotion or continuation of employment. The customary practice in organizations regarding retrenchment when times are bad is based on the concept of “last in, first out,” in which the tenure of service matters (Labuschagne et al., 2005). Research has shown the existence of a negative correlation between continuance commitment and empowerment (Mishra et al., 2016; Murray and Holmes, 2021).

A normative commitment is an individual’s sense of responsibility or obligation. Employees decide to stay within the organization when they feel they must remain there due to a favorable return to the organization or someone who is a referral or a mentor. A sense of obligation pushes employees to stay and not seek out opportunities outside the organization.

Meyer et al. (2004) claimed that organizational commitment comprises affective commitment, continuance commitment, and normative commitment. A meta-analysis by Mathieu and Zajac (1990) concluded that there is evidence of multiple commitments present in an employee. Commitment exists in different forms, such as in organizations, management, supervisors, colleagues, and customers (Becker et al., 1995). Studies attempted to examine its effect on employee attitudes *via* job satisfaction, organizational climate, perceived organizational support, job involvement, job insecurity, productivity, and employee performance (Porter et al., 1974; McMurray et al., 2004; Llobet and Fito, 2013; Rahman et al., 2014; Singh and Gupta, 2014; Vujičić et al., 2015). Meyer and Allen (1997, p. 218) postulated that tri-dimensional models of organizational commitment have been developed and evaluated in Western countries, and their validity and reliability have been established.

Buitendach and De Witte (2005) claimed that job insecurity might evoke feelings of attachment. Research shows that job insecurity decreases commitment among employees (Mowday et al., 1979; Meyer and Allen, 1997; Buitendach and De Witte, 2005). Meanwhile, research has shown that organizational commitment influences various organizational outcomes, such as employee motivation, turnover, absenteeism, and performance. In short, committed employees are likely to perform better (Mowday et al., 1979; McMurray et al., 2004; Buitendach and De Witte, 2005; Vujičić et al., 2015; Shoss, 2017). Therefore, organizational commitment is a behavior that greatly influences productivity and organizational outcomes. It determines employees’ contributions to the organization’s success.

Meyer and Herscovitch (2001) suggested that researchers view commitment as a force that drives employees to take action toward a specific target, with different mindsets shaping their behavior. They claimed that setting the desired outcome, such as performance focus and goal commitment, would address the organizational commitment issue. These perceptions play an important role in encouraging desired behaviors. Past studies suggested that employees were likely to associate with organizations that are perceived as ethical, just, and caring about the welfare of their employees (Chen et al., 2007). According to social exchange theory, people are likely to be reciprocal in their actions. Hence, employees perform better when the organization is “meeting” their expectations. Research has shown that the perception of job insecurity is linked to different negative outcomes, one of which is a decrease in organizational commitment (Mowday et al., 1979; Sverke et al., 2002).

## 6. Positional characteristics

Positional characteristics refer to the terms and conditions the employer and employee mutually agree upon before entering any contract (Employment Act of 1955, 2017). Positional characteristics are also known as the terms of employment, which include employees’ job responsibilities, workdays, hours, breaks, dress code, vacation, sick days, pay, and benefits.

Research has shown that terms of employment have a significant impact on job insecurity and organizational commitment (Orpen, 1993; De Witte, 1999; Tilakdhareem et al., 2010). The following section will elaborate on the literature reviews for the three different variables concerning the terms of employment: flexibility, position in the organization, and tenure in the organization.

### 6.1. Employment flexibility

Due to globalization and rapid changes within the 4th Industrial Revolution, employment flexibility is an exciting factor that draws attention to job satisfaction and job insecurity analysis. It has now become a popular term, especially during the recent pandemic outbreak, when the organization accelerated the transition from traditional deskbound to remote working to ensure business continuity. The employment view has two labor flexibilities: external and internal flexibility. External flexibility refers to regulating the amount of labor by changing the number of people employed. Internal flexibility refers to controlling the amount of labor by adjusting the working time or number of tasks of already-employed workers (Beatson, 1994; Hill et al., 2008; Wilczyńska et al., 2015). In general, the latter, i.e., internal flexibility, is of greater interest. Research has shown that employment flexibility impacts job insecurity (Wilczyńska et al., 2015). The result indicates that the effect of employment flexibility varies across different levels of employees.

### 6.2. Position in the organization

De Witte (1999) argued that there is a curvilinear relationship between job insecurity, job rank, and an individual’s position within the organization (De Witte, 1999). Historically, during times of financial turmoil, manual or blue-collar workers have been among the first to be laid off, while those in managerial roles have been more likely to retain their positions to help the organization navigate the current challenges.

However, a recent study presents a different opinion. Multiple studies confirmed that unemployment is a more severe stressor for managers than for other workers, with unemployed managers showing the same increase in anxiety and depression as unemployed workers (Roskies and Louis-Guerin, 1990).

### 6.3. Tenure in the organization

Research shows a causal relationship between job insecurity and the organization’s service duration (Yousef, 1998; Hellgren and Sverke, 2003). Employees who have been with the organization for a shorter period of time tend to experience more job insecurity than those who have been with the organization for a longer period of time. Newcomers are generally perceived as less stable in their roles as employees and thus more eager to survive within the organization. Apart from that, Labuschagne et al. (2005) claimed that there is a hidden rule of “last in, first out” when it comes to economic turmoil or any unexpected restructuring that occurs within organizations.

## 7. Methods

This paper adheres to Preferred Reporting Items for Systematic Reviews and Meta-Analysis (PRISMA) reporting (Moher et al., 2015). Detailed descriptions are shown in the following section.

### 7.1. Eligibility criteria

**FIGURE 1:**
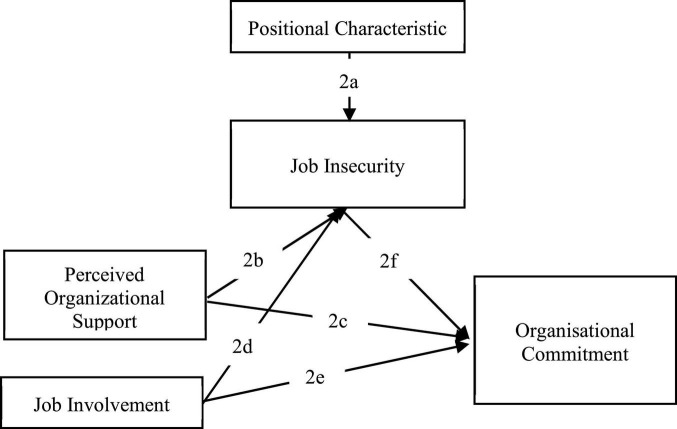
Relationship between variables.

Below are the four criteria for the literature to be eligible for use in this review:

1. Participants: employees are either full-time or part-time workers within a private organization.
2. Measurement: research articles that include any and more of the following, as shown in Figure 1:

a. Job insecurity/security with positional characteristics, employment flexibility, position, and tenure within an organization.
b. Perceived organizational support and job insecurity/security.
c. Perceived organizational support and organizational commitment.
d. Job involvement and job insecurity/security.
e. Job involvement and organizational commitment.
f. Job insecurity/security and organizational commitment.

3. Outcomes: any outcome examining the relationship between constructs presented in criteria 2.
4. Study design: cross-sectional, longitudinal, cross-lagged, or diary study.

### 7.2. Data and search

A total of five databases (Web of Science, PsycINFO, Proquest, PubMed, and ScienceDirect) from the inception of the data up to 31 May, 2021, were included in the study. The search is based on the following keywords:

(a) job involvement and job insecurity/job security;
(b) job insecurity/job security is related to positional characteristics, such as employment flexibility; position within the organization, and tenure within the organization;
(c) perceived organizational support and job insecurity/job security;
(d) perceived organizational support and organizational commitment;
(e) job involvement and job insecurity/job security;
(f) job involvement and organizational commitment;
(g) job insecurity, job involvement, and organizational commitment; and
(h) job security insecurity/job security/perceived organizational support/organizational commitment.

In this study, the article search was limited to research articles written in English. Apart from that, no other restriction was applied. In addition to the results obtained from database searches, the authors also included articles from other sources, i.e., websites and article searches identified through citations.

A comprehensive literature review was conducted in accordance with the proposed framework and the PRISMA guidelines (Moher et al., 2015). Detailed descriptions are shown in the following section.

### 7.3. Data collection

Articles screened at four stages before a decision was included in this paper as a literature review:

1. Articles were screened based on the title and rejected if the title was perceived to be irrelevant to the study.
2. Abstracts were screened and rejected if the abstract from the article was perceived to be irrelevant to the study.
3. A full paper screening to identify whether the article is relevant to the study. At this stage, the authors searched the article’s citation *via* the website. The articles included in the analysis:
a. There are articles searched based on the citations obtained within the literature review, and the data is included in a separate analysis.
b. The articles obtained from citations were analyzed from step 2 onward, followed by steps 3 and 4, before being included in this review.
4. Articles that are eligible based on the criteria mentioned are included in the analysis.

## 8. Search result

**FIGURE 2:**
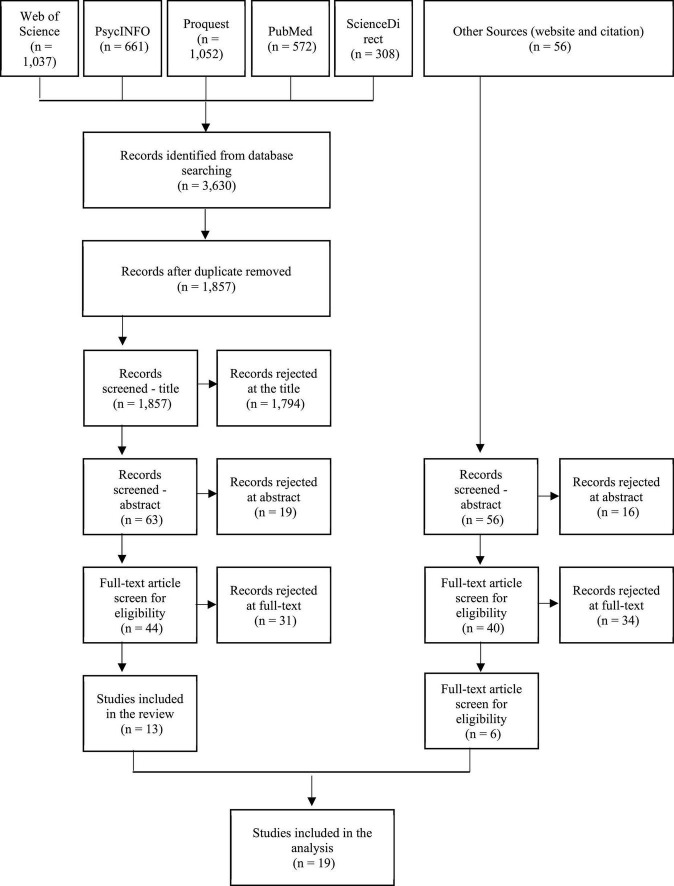
The PRISMA flow diagram of searched, screened, and included studies.

A total of 19 articles were included in the study after performing a PRISMA search. The detailed process is shown in Figure 2. In addition, the summary of the articles is presented in Table 1 for the research details. In summary, the information included the year, the author’s name, the aims, the sample, the location (country) of the study conducted, the design method and result presented, which are related, and the discussion of the article.

**TABLE 1:** Summary of the articles reviewed.

S/N	References	Aims	Sample	Location	Design	Methods	Results	Discussion
1	Ashford et al. (1989)	The article assessed the causes and consequences of JIS using a new theory-based measure incorporating recent conceptual arguments.	183 employees	United States	Cross-sectional	Self-administered *via* self-addressed, stamped envelopes to return the questionnaires	JIS correlates negatively with OC.	Results indicated that personal, job, and organizational realities associated with a perceived lack of control are correlated with measured JIS. JIS, in turn, leads to attitudinal reactions, intentions to quit, reduced commitment, and reduced JS.
2	Rosenblatt and Ruvio (1996)	The study explored the effect of JIS on work attitudes.	385 teachers	Israel	Cross-sectional	The translation was done to Hebrew and administered to teachers	JIS correlates negatively with POS as a consequence JIS correlates negatively with OC.	Results indicated that JIS had an adverse effect on OC, perceived performance, POS, intention to quit, and resistance to change
3	Probst (2000)	Examine JIS by testing hypotheses specifically predict which employees will be impacted the greatest and how these employees will react to the perception of insecurity.	283 employees	United States	Cross-sectional	283 out of 500 employees randomly selected from five agencies, survey administered at the site and site < 10 by emails.	JI correlates positively with JIS JIS correlates negatively with OC.	Perception of JIS has several negative consequences for individuals and organizations: lower job attitudes, decreased organizational commitment, increased health problems, increased psychological distress, and more negative affective reactions to workplace reorganization efforts.
4	De Witte and Naswall (2003)	To analyze whether temporary work and JIS are associated with a reduction in JS and OC.	Belgium—1,120 Netherlands—799 Italy—476 Sweden—476	Belgium, Netherlands, Italy, and Sweden	Cross-sectional	Postal survey	Subjective JIS correlated negatively with OC, except in Sweden, with marginal significant (*p* = 0.07)	The hypothesis was proven in all other countries except Sweden; this could be due to the data collected from 2 organizations during restructuring; as such, the OC is typically low due to uncertainty of employment.
5	Buitendach and De Witte (2005)	Assess the relationship between JIS, JS, and affective OC.	178 maintenance workers	South Africa	Cross-sectional	A representative from different divisions, graded at various levels	JIS correlated negatively with OC	Results showed that JIS correlated negatively with OC, mediated by JS. However, longitudinal research is needed to assess the causal relationship.
6	Adebayo (2006)	The study examined the moderating role of self-efficacy in the relationship between perceived JIS and OC of survivors of retrenchment.	186 employees	Nigeria	Cross-sectional	Self-administered *via* paper and pencil method.	JIS correlates negatively with OC.	Results are consistent with the implications of the social exchange theory and the norm of reciprocity—the role of self-efficacy as a moderator of the JIS-OC relationship.
7	Sora et al. (2010)	It is aimed to study the impact of perceived JIS on employees’ work attitudes and intentions.	942 employees	Spain	Cross-sectional	Self-administered questionnaire under the supervision of the research assistant	JIS correlated negatively with OC	JIS is perceived as a work stressor, which negatively impacts OC. These negative reactions are considered a method for employees to disassociate from the stressor.
8	Blackmore and Kuntz (2011)	Research on JIS as a multidimensional construct; investigate potential antecedents in a restructuring context across contract type.	100 employees	New Zealand	Longitudinal	Self-administered *via* an online survey	POS negatively correlates with JIS	Two dimensional of JIS proven as antecedents, namely positional characteristics (contract type) and environment (POS) Type of contract, emerge as primary contributors to JIS.
9	Peiro et al. (2012)	This article intended to examine the impact of labor conditions on younger employees’ concerns about job loss or JIS and how it relates to their current job performance and the future development of their careers.	3,000 employees	Spain	Cross-sectional	Conducted in 2008, with two stages of sampling and 1,200 interviews Stage 1: Province Stage 2: Municipalities	JI correlates negatively with JIS	These findings provide evidence for the conclusions from previous studies, which claimed JIS is a work stressor with detrimental consequences for employees.
10	Ngo et al. (2013)	This article aimed to examine the mediating role of organizational identification in the relationship between employees’ feelings of organizational context and their job attitudes.	591 workers	China	Cross-Sectional	Self-administered questionnaire	POS correlates positively with affective OC Perceived JIS correlates negatively with affective OC	Organizational identification is positively associated with affective OC and JS and negatively associated with intentions to leave. Turning to antecedents, POS enhances organizational identification while perceived JIS reduces.
11	Ngo et al. (2013)	The authors examined the effects of perceived JIS and psychological capital on employees’ OC and JS.	257 employees	Hong Kong	Cross-Sectional	Self-administered questionnaire	JIS correlates negatively with OC.	Results were obtained similarly with the Western country. Resilience attenuates perceived JIS’s negative impact on attitudinal outcomes (OC and JS).
12	Marques et al. (2014)	It is aimed to analyze the simultaneous effects of perceived JIS and OC on the innovative behavior of workers in an announced downsizing environment.	88 valid returned surveys	Portugal	Cross-Sectional	Self-administered questionnaire.	JIS correlates negatively with OC.	JIS negatively correlates with workers’ attitudes, and results showed a negative relationship between JIS and OC. The result further revealed that OC might mediate the relationship between JIS and innovative behavior in a downsizing organization.
13	Istonova and Fedakova (2015)	Aim to compare Slovakia and Estonia’s employees’ job insecurity by looking at socio-demographic, job, and organizational predictors and individual and social consequences	Estonian–547 Slovak—530	Slovakia and Estonia	Cross-Sectional	European Social Survey	JIS correlates with employment type	Results show that the type of contract was significantly associated with a higher level of job insecurity in both countries. However, regression analyses did not confirm the types of contracts as a significant predictor of job insecurity, neither for Estonia nor Slovakia.
14	Urbanaviciute et al. (2015)	This paper analyses job insecurity with psychological contract breach and focuses on employee attitudes, such as JS and organizational commitment within the framework of stress theories and	1,180 employees	Lithuanian	Cross-sectional	Online questionnaire	Qualitative JIS correlates negatively with OC.	The deterioration of employee attitudes in response to job insecurity can be attributed to withdrawal reactions, a well-known phenomenon in stress research, contributing to employee attitudes. Threats to favorable working conditions may trigger withdrawal reactions.
15	Vujičić et al. (2015)	This article analyses the relationship between JIS, JS, and OC among employees	149 employees	Serbian	Cross-sectional	Self-administered *via* paper and pencil method.	JIS correlates negatively with OC.	A negative correlation between JIS, JS, and OC variables indicates that employees who do not feel secure and are less committed to the company, contrary to earlier studies of workers with temporary jobs who perceive their job as more insecure.
16	Frone (2018)	Explored the effects of the Great Recession on workers who remained employed; to assess net population change in JIS and employment insecurity, physical and mental health, and effective OC.	Before the recession (*N* = 2,354) during the recession (*N* = 2,322)	United States	Longitudinal	National Survey of Workplace Health and Safety. Randomized telephone survey from the target population.	JIS correlates negatively with affective OC.	The present findings provided initial support by showing that JIS mediated the association of the Great Recession to physical health, mental health, and organizational commitment.
17	Bose and Biswas (2019)	It aimed to compare the job security perceptions between the managerial and non-managerial sectors and between the Emirate nationals and expatriates.	280 employees	Dubai	Cross-sectional	Self-administered questionnaire	Hypotheses not supported. The level of job security perceptions was found not significant with employee positions.	The level of JIS perception is not significant and further reveals that the factors like employment scenario, the scope of growth and learning, employers’ compliance with rules, and organizational practice play significant roles in influencing JIS perceptions of the employees in Dubai.
18	Pattnaik et al. (2020)	This article studies the relationship between POS, person– Organization fit (P–O fit) and OC. It highlights the importance of providing organizational support to employees To foster their commitment to the organization.	430 employees	India	Cross-sectional	Self-administered questionnaire	POS correlates positively with affective OC	The study established the potential moderating role of P–O fit in the relationship between POS and OC.
19	Stankeviciute et al. (2021)	This article intended to discover the linkage between JIS and happiness at work and its dimensions, namely JS, affective OC, and work engagement	350 questionnaires	Lithuanian	Cross-sectional	Paper questionnaire distributed to employees; results collected within a month.	JIS correlates negatively with affective OC.	The perceived threat of losing the job is related to lower levels of employees’ emotional attachment to involvement and identification, which is supported by meta-analysis.

JI, job involvement; POS, perceived organizational support; JIS, job insecurity; OC, organizational commitment; JS, job satisfaction.

There are supposed to be six subsections of empirical review, as listed below. However, SLR yielded no results in the search for articles that studied job involvement and organizational commitment (item 5). Hence, the SLR analysis only reflects on five of the remaining sections.

1. Job insecurity with positional characteristics.
2. Perceived organizational support and job insecurity.
3. Perceived organizational support and organizational commitment.
4. Job involvement and job insecurity.
5. Job involvement and organizational commitment.
6. Job insecurity and organizational commitment.

## 9. Finding and discussion

Out of the 19 articles, two were longitudinal study designs, while the rest were cross-sectional studies. Most of the research was aged more than a decade and five within 5 years of this article. All of them are self-administered questionnaires, either by hard copy or online survey, except one study that uses a randomized telephone survey. All papers presented were published in English, one of the search criteria.

The comprehensive SLR yielded 19 articles that provide insights into the relationship between job involvement, perceived organizational support, organizational commitment, and job insecurity. Most studies were conducted in western countries, and five out of nineteen were from Asia; however, none were from Southeast Asia. Table 2 provides a summary of the countries and continents mentioned in the articles. Hence, the result may or may not be able to be generalized across Southeast Asia or Malaysia specifically.

**TABLE 2:** Summary of countries and continents.

Countries and continents	Articles	
African		**2**
Nigeria	1	
South Africa	1	
Asia		**5**
China	1	
Dubai	1	
Hong Kong	1	
India	1	
Israel	1	
Europe		**8**
Belgium, Netherlands, Italy, and Sweden	1	
Lithuanian	2	
Portugal	1	
Serbian	1	
Slovakia and Estonia	1	
Spain	2	
North America		**3**
United States	3	
Oceania		**1**
New Zealand	1	

An interesting finding in the relationship between job insecurity and organizational commitment is shown in Table 1, S/N 4. During the recession, the significant impact on organizational commitment has shown to be only marginal (De Witte and Naswall, 2003). During the organizational restructuring, employees may experience a high level of uncertainty about their job status, regardless of their current level of job insecurity. This is because they may not know what will happen as a result of the restructuring and may be concerned about the possibility of losing their job. Therefore, it is important for organizations to consider the impact of restructuring on their employees and to support them through these uncertain times, as indicated by the Conservation of Resources Theory. Employees will conserve energy by committing to the job when they feel stressed, focusing on new resources or employment. The relationship between job insecurity and organizational commitment has been widely discussed and paired with other constructs, such as happiness at work and job involvement. Researchers are interested in finding additional linkage and information among the constructs to enable human resources practitioners to identify employees’ attitudes, enhance productivity, and, at the same time, reduce absenteeism.

Most of the research articles (14) focused on the relationship between job insecurity and organizational commitment as consequences, and limited studies were conducted on the antecedents of job insecurity. It proves that job insecurity is widely recognized as the cause of lower organizational commitment, although it has been posited as an indirect variable. Organizational commitment improves productivity and compensates for the time lost when employees are less committed to the job. They typically spend time and effort on other matters, such as looking for alternatives or dealing with high absenteeism. Thus, it also leads to higher medical costs, attrition, and loss of knowledge during the transition. Table 3 provides a quick summary of the analysis based on the articles presented.

**TABLE 3:** Summary of the analysis between constructs.

Analysis	Articles
Job insecurity and organizational commitment*	14
Job insecurity and positional characteristics	2
Job involvement and job insecurity*	2
Perceived organizational support and job insecurity	2
Perceived organizational support and organizational commitment*	2

*Refer to Table 1, Article S/N 2 and 3, with more than one hypotheses.

Job involvement and perceived organizational support are essential elements because employees’ resources support them to excel in their jobs and remain competent at work. Job involvement refers to the importance that an employee places on their job and the extent to which they feel invested in it. When an employee is highly involved in their job, they are more likely to contribute and be engaged in their work. Perceived organizational support measures the opposite of such commitment, where it measures the organization’s commitment toward employees, such as appreciation, wellbeing, and satisfaction on the job. Some of the research studied the influence of perceived organizational support on organizational commitment. Researchers view it as a reciprocal relationship where one has to feel supported, and in return for the favor, one will commit to the job. In the past, job insecurity was studied as a consequence of perceived organizational support: when employees perceive that they are not receiving sufficient support from their organization, they may feel insecure (Rosenblatt and Ruvio, 1996). Researchers may consider exploring the potential negative impacts of these constructs.

Technology has transformed the organizational landscape in line with the 4th Industrial Revolution. In many cases, employees do not need to be physically present in an office setting to perform their duties, with the exception of certain roles that require operations or deskbound jobs. An SLR discovered that none of the articles examined employment flexibility, tenure, or position within the organization as potential factors that could impact job insecurity. The old saying “out of sight, out of mind” may not be relevant in contemporary society. However, organizations must ensure that employees feel engaged, even though they do not need to physically sit in the office or practice flexible working hours, days, or locations. For example, they are working from home or in a café instead of in the office. Remote work will essentially reduce commute times and, at the same time, ease traffic conditions, especially during peak hours. How the new norms impact job involvement, job insecurity, perceived organizational support, and organizational commitment remains unknown.

Therefore, given the exploration of the relationship between the constructs, the aforementioned could be a potential direction of future research to study the antecedents of job insecurity and the potential mediator role that job insecurity plays. Practitioners must identify the root cause of insecurity to prepare a mitigation plan. For example, to retain the current employee and attract potential and future employees.

### 9.1. Empirical studies of positional characteristics and job insecurity

There is limited literature that relates job insecurity with the positional characteristics of employment flexibility, position, and tenure within the organization. Current research is skewed toward the mediation relationship between job insecurity and other variables such as job involvement, perceived organizational support, and organizational commitment (Adebayo, 2006; Blackmore and Kuntz, 2011; Ngo et al., 2013; Bohle et al., 2018). Of the available literature, contrary to the prediction by Shoss (2017), Bose and Biswas (2019) concluded in their study that position within the organization has no significant relationship with job insecurity.

It is crucial to explore and study whether positional characteristics, particularly employment flexibility, impact job insecurity. This is especially relevant during the current pandemic when many employees are working from home or organizations have officially moved from traditional working arrangements to the “hot seat,” in which employees are no longer assigned to a specific work desk but instead share desks with others.

### 9.2. Empirical studies of perceived organizational support and job insecurity

The feeling of attachment and belonging develops over time (Mowday et al., 1979). Therefore, a typical recruit in an organization will have a neutral sentiment. However, some will have certain expectations due to the information they obtained regarding the organization before their employment. Thus, it is safe to say that employee behaviors and attitudes are shaped by their experiences within the organization over time. The events and circumstances they encounter during their tenure can have a lasting impact on their behaviors and attitudes. Therefore, organizations should be aware that any policy or standard operating procedure directly impacts the development of employees’ perceptions and the consequences if the said perception falls on the opposing side.

The literature revealed a negative correlation between perceived organizational support and job insecurity. Rosenblatt and Ruvio (1996) concluded that perceived organizational support is the consequence of job insecurity, whereas Blackmore and Kuntz (2011) found that perceived organizational support is the antecedent to job insecurity. The two variables are interchangeable, given the causes and effects. Alternatively, it could be an indication of a reciprocal relationship. Apart from this, both articles have a different “direction” given the relationship between perceived organizational support and job insecurity; researchers placed perceived organizational support as the moderator. Bohle et al. (2018) concluded that perceived organizational support moderates job insecurity and organizational commitment. In a way, it has been proven in past literature that no single factor or variable influences overall organizational behavior. Instead, it is a combined issue that influences employee behavior and productivity.

It is interesting to explore the relationship between perceived organizational support and job insecurity. Thus, this review focuses on the few possible antecedents of job insecurity and tests job insecurity in the framework proposed by Shoss (2017) on the relationship between job involvement, perceived organizational support, job insecurity, and organizational commitment.

### 9.3. Empirical studies of perceived organizational support and organizational commitment

Perceived organizational support focuses on the reciprocal relationship where employee and employer contributions count (Eisenberger et al., 1986; Rhoades and Eisenberger, 2002). Finally, organizational commitment is a positive attitude and a psychological connection with the organization that generates a positive environment within the employee and possibly influences the colleagues around them (Mowday et al., 1979).

Eisenberger et al. (1986) linked organizational commitment to the behaviors of employees, such as absenteeism and medical costs. They concluded that employees with a strong exchange ideology, which is in line with the social exchange theory, exhibit a higher percentage of absenteeism. Ngo et al. (2013) found that perceived organizational support enhances organizational identification and influences employees’ job attitudes, such as affective organizational commitment. Both studies have concluded that perceived organizational support positively impacts organizational commitment.

Perceived organizational support is the perceived level of support employees feel from their organization. It can increase when employees feel appreciated or acknowledged. Conversely, it decreases when employees feel demotivated or unsupported in terms of work or work-life balance. The systematic literature review indicated the need for further research on the relationship between perceived organizational support and organizational commitment and their impact on productivity and morale within an organization.

### 9.4. Empirical studies on job involvement and job insecurity

Although the study of job involvement has been available for the past few decades (Brown, 1996), there are limited studies examining the relationship between job involvement and job insecurity (Shoss, 2017). Current literature mainly focuses on the relationship between job involvement and other variables, such as organizational commitment and job satisfaction, either as mediating or moderating factors (Clay-Warner et al., 2005; Singh and Gupta, 2014; Mendoza, 2019; Salessi and Omar, 2019).

While conducting the systematic literature review, the articles revealed contrary research findings on the relationship between job involvement and job insecurity. For example, Peiro et al. (2012) concluded that job involvement is negatively correlated with job insecurity, which is detrimental to employee wellbeing and performance. In contrast, Probst (2000) research showed that job involvement was positively associated with job insecurity. The argument was that when an employee is highly involved in their job, they may have a greater fear of losing it compared to those with lower levels of involvement. As such, this could lead to a curvilinear relationship between job involvement and job insecurity.

Interestingly, two different views were found in the literature review. First, Peiro et al. (2012) shared a similar concept with Shoss (2017), where employees feel lower job involvement at work, which triggers job insecurity. However, Probst (2000) claimed that a highly involved worker may experience high job insecurity due to the amount of time their work occupies. However, according to the SLR, we can conclude that there are limited studies on the relationship between job involvement and job insecurity, as well as the potential implications of job involvement on job insecurity.

### 9.5. Empirical studies on job insecurity and organizational commitment

Organizational commitment is a consequence that arises from an employee’s experiences with various parties, such as the organization, colleagues, supervisors, and external parties. Hence, researchers focused on the influence of organizational commitment toward productivity and the impact of job insecurity on organizational commitment (Ashford et al., 1989; Rosenblatt and Ruvio, 1996; Probst, 2000; De Witte and Naswall, 2003; Buitendach and De Witte, 2005; Adebayo, 2006; Sora et al., 2010; Ngo et al., 2013; Marques et al., 2014; Urbanaviciute et al., 2015; Vujičić et al., 2015; Frone, 2018; Stankeviciute et al., 2021).

Literature reviews from Asia (Hong Kong, China, and Israel), Africa (Nigeria and Serbia), Europe (Belgium, Netherlands, Italy, Spain, Portugal, Lithuania, and Serbia), and the United States of America shared the same outcome: job insecurity negatively correlated with organizational commitment (De Witte and Naswall, 2003). With Sweden being the exception, the authors suggested that job insecurity and organizational commitment are typically low during organizational restructuring. Therefore, the result can be generalized worldwide, as no cultural differences exist. In conjunction with the Conservation of Resources Theory, job insecurity influences organizational commitment. Employees will reserve resources (commitment) when they possess them under threat (job security equates to financial status).

Literature reviews revealed that the relationship between job insecurity and organizational commitment is mediated by several factors, such as positional characteristics (Blackmore and Kuntz, 2011), self-efficacy (Adebayo, 2006); psychological capital (Ngo et al., 2013), and perceived organizational support (Bohle et al., 2018).

## 10. Theoretical contribution

This study significantly contributes to the existing body of knowledge and framework by examining the relationship between job insecurity, job involvement, and perceived organizational support as antecedents and organizational commitment as consequences. Although there are numerous studies on the aforementioned variable, only a few of them have examined the relationship between the four variables in a single research study, as previously noted. Furthermore, research on the relationship between job involvement and organizational commitment is scarce. Therefore, the research would extend the theoretical perspective on the relationship between job involvement, perceived organizational support, job insecurity, and organizational commitment. Therefore, this study focused on job insecurity, its antecedents, mediation consequences, and how it affects organizations.

## 11. Practical implication and future research

This literature review provided a few practical implications. First, the findings suggested that although job insecurity has been widely studied in the past, none of them analyzed both antecedent variables and consequences in the same study. Shoss (2017) has introduced a comprehensive framework for explaining the potential antecedents and consequences influencing productivity. The conceptual model presented antecedents from various angles, such as micro- and macro environment, individual and organizational perspectives, threats, and consequences. This study examines research articles as an extension of the conceptual model presented by Shoss.

Practitioners and business owners are interested in understanding how to enhance employees’ productivity, and one way to do so is by providing fringe benefits such as gym access to promote work-life balance. Therefore, future studies should explore the antecedents and consequences of job insecurity and possible mediation or moderation role. Putting a focus on the impact of employment flexibility on job insecurity, particularly given the shift toward work and other flexible employment arrangements in the wake of the pandemic.

Secondly, most of the studies focused on western countries and cultures, which makes it challenging for the local practitioner in Malaysia to make generalizations and direct comparisons. The study of the relationship between job insecurity, job involvement, and perceived organizational support and how job insecurity impacts organizational commitment enables the organization to tackle the issue of high attrition and low organizational commitment among the employees (Van Hootegem et al., 2019). Apart from the attrition rate, absenteeism is one of the most critical elements from a financial standpoint.

Third, more research is needed to relate job insecurity to the differences in the positional characteristics believed to play a part in job insecurity. With the recent pandemic outbreak, most organizations have to change their landscape and working culture. Furthermore, the Ministry of Human Resources Malaysia has introduced flexible working arrangements in terms of flexible working hours, days, and places under the latest amendment of the Employment Act of 1955 (Tan, 2022). Hence, research on the influence of employment flexibility on job insecurity and other positional characteristics, such as organizational tenure, should be studied. The global or local lockdown forced organizations to focus on cost rationalization, and retrenchment became unavoidable. According to the “last in, first out” rule, an employee’s length of service, or tenure, is often a key factor in determining employment security, as those with longer tenure are less likely to be laid off or have their contracts terminated.

Future research could consider the positional characteristics of employees as a way to add value to empirical studies; this could allow organizations to develop different policies and procedures for handling employees at different levels or with varying lengths of tenure, potentially as a means of rewarding loyalty. As shown in Table 3, the potential antecedents that cause job insecurity have yet to receive enough attention from researchers. Hence, more research should be done to identify the possible root causes of job insecurity and the relationships between the variables.

## 12. Limitations

There are many constructs related to job insecurity, such as job satisfaction, the intention to leave, and burnout, to name a few, which can act as both antecedents and consequences. However, this paper only examined the few key constructs identified by the authors: job involvement and perceived organizational support as antecedents and organizational commitment as a consequence of job insecurity. Future research could expand upon this by exploring more variables to provide a more comprehensive literature review.

The paper excluded moderator or mediator analysis. However, some variables could function as moderators, if not mediators, between the relationships mentioned. For example, perceived organizational support could be a potential moderator between job insecurity and organizational commitment. Hence, future literature reviews could potentially focus on moderation and mediation in the study.

By conducting a systematic literature review *via* the PRISMA guidelines, this study demonstrated a clear and concise method, as shown in the flow chart. However, this study chose five primary sources for data searches: Web of Science, PsycINFO, Proquest, PubMed, and ScienceDirect. Fifty-six articles were identified through the initial search, with links obtained from the sources cited. Of these, 19 articles were included in the analysis, six of which were obtained through citations, accounting for 30% of all articles. Hence, expanding the sources of data search could potentially yield significant results in an SLR.

## 13. Conclusion

Studying the antecedents and consequences of job insecurity is crucial for practitioners to develop strategies for managing human resources processes and improving organizational productivity. Retaining talented employees and reducing voluntary attrition is important for organizations, as it is often more cost-effective to retain current employees who have assimilated into the company culture and have gained valuable job knowledge than to constantly recruit and train newcomers. By addressing issues of job insecurity through methods such as increasing job involvement and perceived organizational support, organizations can reduce employee turnover and ultimately enhance productivity. Therefore, researchers must explore the factors that contribute to increased productivity and sustainability.

## Data availability statement

The original contributions presented in this study are included in the article/supplementary material, further inquiries can be directed to the corresponding authors.

## Author contributions

CH contributed to the conceptualization and prepared the SLR data analysis and manuscripts with N-AA supervision. WW and NZ were involved in conceptualization and supervision. All authors listed have made significant contributions in critically reviewing and approving the final version for publication.
